# Applications of Life Cycle Assessment in the Chocolate Industry: A State-of-the-Art Analysis Based on Systematic Review

**DOI:** 10.3390/foods13060915

**Published:** 2024-03-18

**Authors:** Shuhan Wang, Yahong Dong

**Affiliations:** 1 Macau Environmental Research Institute, Macau University of Science and Technology, Macao, China; hannnsu@163.com; 2 National Observation and Research Station of Coastal Ecological Environments in Macao, Macau University of Science and Technology, Macao, China

**Keywords:** chocolate, cocoa, environmental impact, life cycle assessment, review

## Abstract

Chocolate is a popular food for its unique flavor and taste, rich nutritional value, and the psychological values brought to people. The raw material production of chocolate, product manufacturing, sales and transportation have different degrees of environmental impact. This review explores the environmental hot spots in the life cycle of chocolate and puts forward corresponding suggestions for the improvement. By applying a systematic review method, this paper collected 25 articles on life cycle assessment (LCA) of the environmental impact of the chocolate industry. It is found that the life cycle of chocolate has the highest environmental impact in the raw material production and chocolate manufacturing stages (accounting for 77–97% of total impacts), among which milk powder, sugar and cocoa derivatives are the important contributors to significant environmental burden. Dark chocolate generates the lowest carbon emissions (1.67 kg CO_2_ eq/kg product) among existing chocolate categories, while the chocolate confectionery products release the highest carbon emissions (6.76 kg CO_2_ eq/kg product) among chocolate-containing products. Improvement measures are proposed for reducing environmental impacts and for selecting environmentally friendly product formulae. This study can provide benchmarking for the chocolate industry and improves the understanding of life cycle environmental impacts of chocolate products.

## 1. Introduction

Chocolate is a popular food product that holds cultural, social and economic significance. The history of chocolate can be dated back to ancient times, when chocolate and its main ingredient cocoa were first used as a therapy and drug carrier [1]. The appreciated taste of chocolate, enhanced by mixing with cassia bar and pepper [2], as well as sugar, vanilla, and others, has spurred its evolution into multiple variations like beverages, confectionery, and other delightful creations. Worldwide, chocolate is mostly consumed in European countries, such as Switzerland, Austria, and Germany [3]. The prominent exporting nations are Belgium and Italy, while the United States, France, and the Netherlands are the leading importing countries [4]. In 2021, the annual consumption of chocolate was 8.13 million tons [5], and the global market size was USD 113.6 billion, with an expected annual growing rate of 3.7% towards 2030 [6], demonstrating the chocolate industry can anticipate persistent growth in future.

Despite its delectable treats, the production of chocolate involves various processes that have been accused of causing environmental degradation. The cultivation of cocoa beans, the main ingredient in chocolate, has led to deforestation and habitat destruction in tropical forests [7], imposing challenges to the sustainable development of the industry [8] in the main cocoa production countries like Ghana and Indonesia [9,10]. In addition, life cycle processes of chocolate can consume considerable energy [11], especially in the mixing process [12], making energy conservation a matter of importance for chocolate manufacturers [13]. The manufacturing processes of chocolate can give rise to air and water emissions, and generate liquid and solid wastes [14]. Given the inevitable environmental impacts caused by the chocolate industry, it is imperative to evaluate their effects and explore sustainable practices within the industry.

Life cycle assessment (LCA) has been widely adopted as an important instrument in evaluating the environmental performance of a product or service from the “cradle to grave” [15,16]. Existing studies have implemented LCA to analyze the environmental impacts of chocolate products [17], demonstrating that LCA is an effective and efficient environmental assessment method for the selection of environmentally friendly management strategies in the industry. However, there is a significant lack of research reviewing the application of LCA in the chocolate industry. This knowledge gap creates obstacles in comprehending the industry’s environmental performance across its life cycle, thereby puzzling researchers and industrial professionals seeking to facilitate a sustainable transition.

In order to fill the research gap, this study aims to investigate the state-of-the-art LCA applications in the chocolate industry based on a systematic review of twenty-five existing case studies. First, the research trends of the reviewed articles are explored to unveil their geological and temporal distributions through a bibliometric analysis. In the next section, a critical review of the selected studies is carried out following the four phases of LCA, namely, goal and scope definition, life cycle inventory, impact assessment, and interpretation. Statistics on data inputs and assessment results are analyzed and summarized. The environmental performances of different chocolate types are compared through both quantitative and qualitative approaches. Lastly, limitations and future opportunities are discussed regarding the assessment methods and the sustainable industrial practices.

## 2. Material and Methods

### 2.1. Description of Systematic Review

A systematic review collects and synthesizes results from selected studies to solve specific research questions with predetermined goals [18,19]. Few studies have adopted systematic reviews to examine and summarize existing LCA studies in the food and agricultural industries [20,21], while the systematic review has been recognized as a reliable and effective review method. In general, five steps are involved in a systematic review, including framing questions, identifying relevant work, assessing the quality of studies, summarizing the evidence, and interpreting the findings [22,23].

### 2.2. Framing Questions

It is critical to raise research questions for a systematic review, in a structured and explicit manner [22]. This study is the first attempt to conduct a systematic review that explores the state-of-the-art development of LCA implementations in the chocolate industry. Therefore, the first research question is (i) What is the state-of-the-art development of LCA studies in the chocolate industry? Following the typical four phases of LCA, this study investigates the critical issues in each phase to unveil the findings of chocolate LCA studies and the challenges in detail. The second research question is (ii) What are the findings and challenges of LCA studies on the chocolate industry? Lastly, the best practices in terms of life cycle environmental performance should be identified to provide recommendations for the future sustainable development of the chocolate industry. The third research question is (iii) What are the recommendations for the chocolate industry in reaching towards sustainable development?

### 2.3. Locating the Studies

The search of LCA case studies was carried out using well-recognized databases, including Science Direct, Springer, and Web of Science. The search string was (“Life cycle assessment” or “LCA”) and (“chocolate” or “cocoa”). Due to the small number of articles on the LCA of cocoa and chocolate before 2000 and their lack of relation to the research topic, the studied period was confined to 2000 to 2022. The scope of the search included titles, abstracts and keywords. The searching process started on 8 December 2022 and the last search was completed on 16 February 2023. The initial search resulted in 46 articles for the next step of screening.

### 2.4. Selection and Evaluation

**Figure 1:**
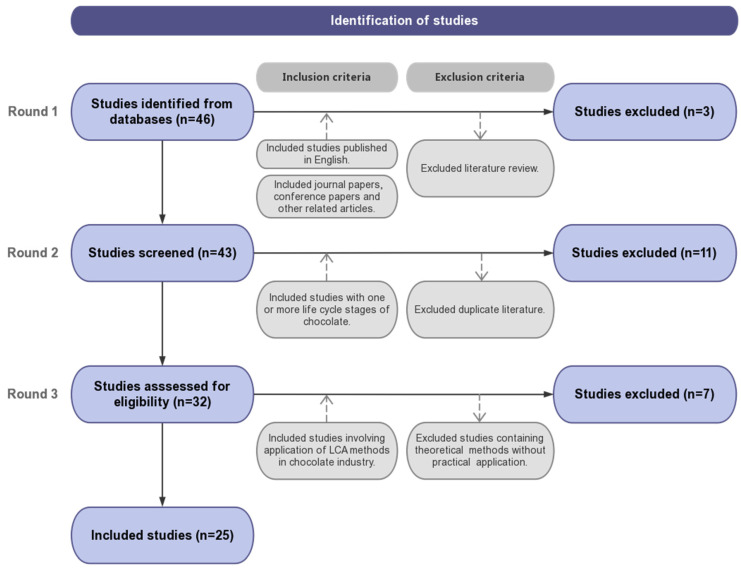
The screening process to select LCA case studies from the chocolate industry.

The screening process was primarily based on the widely adopted Patient, Intervention, Comparison, Outcome (PICO) framework, used to construct criteria of intervention review eligibility [24]. The inclusion and exclusion criteria were set to determine the validity of the systematic review results [25]. Figure 1 gives the selection procedures. The first round examined the language and study types. We only included articles published in English to ensure a comprehensible analysis. Review articles were excluded as this review focused on LCA case studies of chocolate. Three articles were excluded in Round 1. The second round sought to guarantee the quality of the selected literature. By further examining the contents of articles, case studies that involved at least one life cycle stage of chocolate were included. Duplicate publications due to overlapping data, methods, and conclusions can result in not only ethical and legal problems, but also negative impacts on research [26]. Therefore, duplicate articles were excluded. In Round 2, 11 articles were excluded. The last round sought to conduct a detailed review of the selected articles to exclude studies that only focused on theoretical methods without the applications of these methods in a case study, and 7 articles were excluded. Consequently, 25 articles were selected for the review analysis (Appendix A).

## 3. Results

### 3.1. Publication Trend

**Figure 2:**
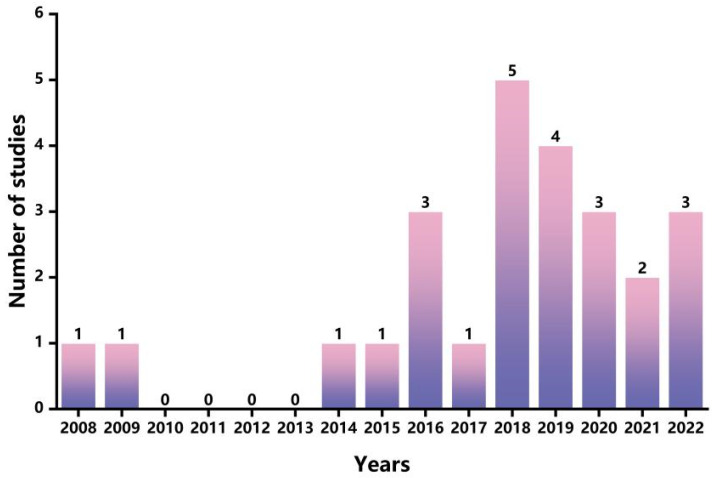
Yearly distribution of selected LCA case studies in the chocolate industry.

**Figure 3:**
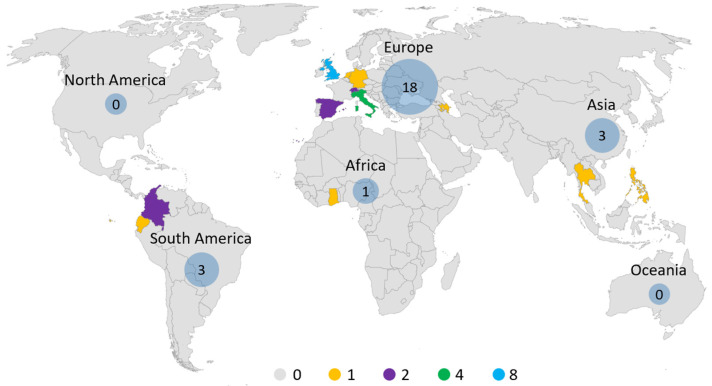
Spatial distribution of selected LCA case studies in the chocolate industry.

The numbers of publications from 2008 to 2022 are shown in Figure 2. Apparently, more publications were observed after 2016, with a peak of five articles in 2018, indicating applications on LCA in the chocolate industry will attract increasing attention in the future. Figure 3 shows the spatial distribution of selected articles. The majority of the literature was published in Europe, with the United Kingdom publishing eight articles, followed by Italy, which published four articles. Asia and South America each published three articles. There was only one article published in Ghana, Africa. However, no publications on the LCA of chocolate were found in North America or Oceania. Hence, there is a limited amount of published research on LCA for the chocolate industry, both in terms of quantity and geographical distribution. There is a need to consolidate existing studies on LCA in the chocolate industry and pave the way for future directions in chocolate LCA research.

### 3.2. Goal and Scope Definition

#### 3.2.1. Study Aim

**Figure 4:**
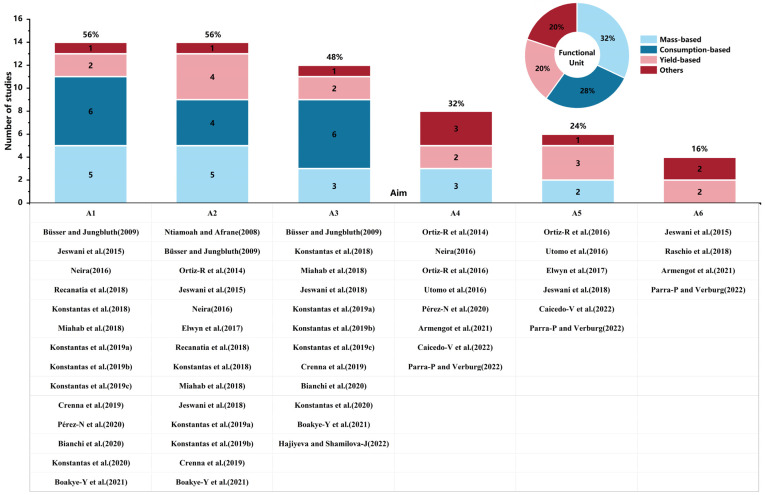
Study aims and functional units of the LCA studies in the chocolate industry.

As shown in Figure 4, there are six aims of LCA studies performed in the chocolate industry: (A1) to identify significant processes, (A2) to assess the life cycle’s environmental impacts, (A3) to compare the environmental impacts of different products, (A4) to compare the environmental impact of a specific stage under different design conditions, (A5) to focus on certain life cycle stages, and (A6) to integrate LCA with other techniques. The first three aims (A1–A3) have been most widely studied. In addition, many articles focused on more than one aim (e.g., [27]).

It was found that 14 studies (56%) focused on A1 in order to identify significant processes. For example, Recanati et al. [19] evaluated the environmental performance of dark chocolate, identified the hotspots of emissions throughout the supply chain, and found that raw material production, transportation, and manufacturing are the most significant processes. A2 regards the intention to assess the life cycle environmental impacts, which was the focus of 14 studies (56%). For instance, Pérez-Neira et al. [17] evaluated the “cradle-to-grave” life cycle stages of dark chocolate, including production, transportation, manufacturing, retailing, and disposal. A3 was the aim of 12 studies (48%). For example, Boakye-Yiadom et al. [28] compared extreme dark chocolate (EDC, with 72% cocoa percentage), dark chocolate (DC, with 56% cocoa percentage), milk chocolate (MC, with 38% cocoa percentage), and flavored milk chocolate (FMC, with 38% cocoa percentage). Their results show that DC performed the best in most of the impact categories, while FMC demonstrated the greatest environmental impacts. A4 was studied by eight articles (32%). For example, Armengot et al. [29] compared four cocoa production systems, i.e., agroforestry under conventional management (CA), agroforestry under organic management (OA), full-sun monoculture under conventional management (CM), and full-sun monoculture under organic management (OM). A5 evaluates certain life cycle stages. James et al. [30] evaluated cocoa production and primary processing stages, and found that transportation, harvesting, pest management, and nutrient management contributed the most to environmental impact categories. A6 integrates LCA with other techniques. For example, Parra-Paitan and Verburg [31] conducted an attributional LCA and integrated it with land use modeling and spatial analysis to explore the environmental impacts.

#### 3.2.2. Functional Unit

A functional unit (FU) is a quantified measure of the performance of a product system that serves as a reference unit [32]. The FUs that were adopted by the reviewed articles can be categorized into four types, i.e., mass-based, consumption-based, yield-based, and others (Figure 4).

It was found that eight studies (32%) used mass-based FU. This type of FU can be adopted to calculate emissions and energy consumption from cultivation to manufacture/sale by analyzing a certain mass of chocolate products. For the studies that focused on cocoa cultivation stage, a certain quality of dried cocoa beans or pods was adopted as the FU [33,34]. Seven studies (28%) adopted consumption-based FUs, such as 1 kg chocolate biscuits consumed at home [35]. The studies that adopted this type of FU mainly focused on the life cycle stages of distribution by retailers and consumption by customers. In addition, the consumption-based FU can refer to consumption by individuals, households, sectors, or countries. Five studies (20%) used the yield-based FU, such as 1 kg of packaged candy products [36]. This type of FU was adopted by studies to conduct LCA mainly for the manufacturing stage. In addition, there are other types of FU that were used in the reviewed studies. For example, Bianchi et al. [37] adopted both 1 kg and 1 kcal of chocolate as FUs. Caicedo-Vargas et al. [38] used 1 kg of cacao, 1 kg of sold products, and 1 hectare for their economic analysis. Ortiz-R et al. [27] also used 1 hectare of cocoa farming land as an FU. Therefore, the first three types of FUs are based on a certain amount of mass and were adopted by most of the reviewed studies. The FU should closely reflect the function of the studied object, and it is essential to first determine the market sector and obligatory product properties [39]. The functional unit of food products should prioritize relevance to mass or volume rather than land use [40], while a more accurate functional unit selection would account for both nutritional composition and quality, offering solutions to existing by-product distribution issues in certain foods and better reflecting their functions [41].

#### 3.2.3. System Boundary

**Figure 5:**
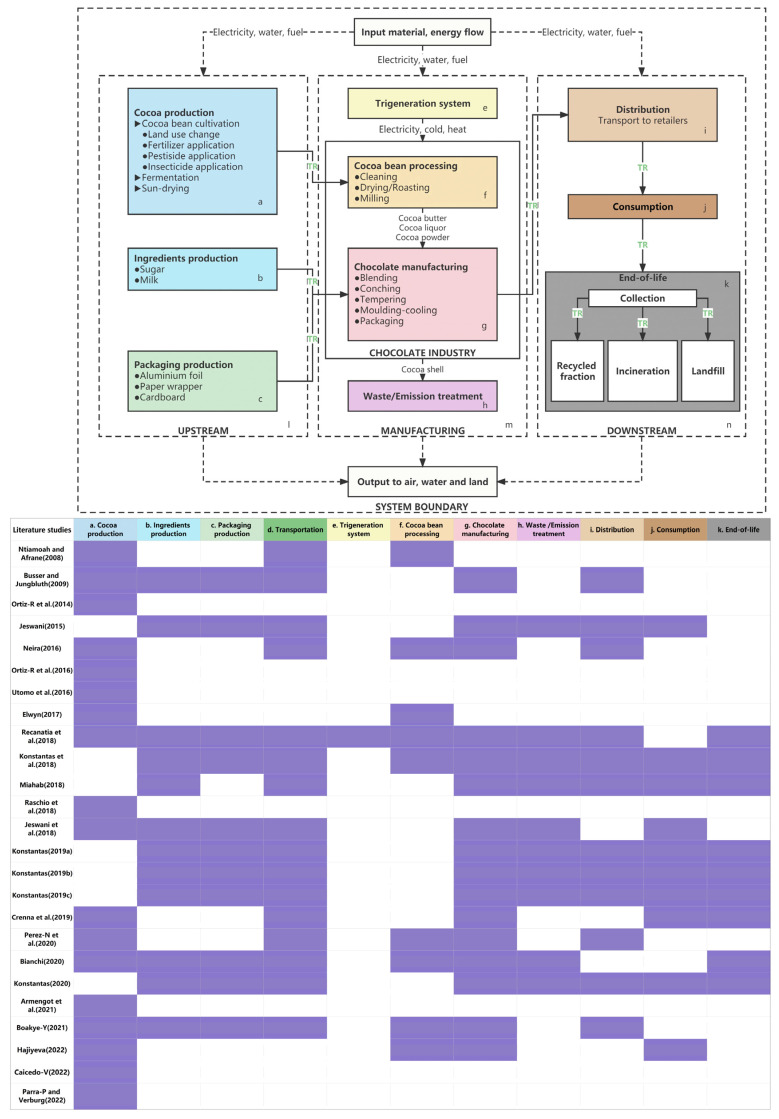
System boundaries of LCA studies of the chocolate industry. The life cycle stages are distinguished by different colors and denoted with letters. TR refers to transport. The life cycle stages that were covered by the reviewed studies are shaded in purple.

The life cycle stages of chocolate can be generally categorized into three phases, i.e., upstream, manufacturing, and downstream phases [19,28]. As shown in Figure 5, the life cycle of chocolate begins with the cultivation of cocoa, the acquisition of other ingredients, such as sugar and milk, as well as the production of packaging materials [28,35,42,43,44]. After harvesting, the cocoa beans are fermented and dried under sunlight [37], and then delivered to the chocolate factory to be treated under the trigeneration system [19], including processes of cleaning, roasting, and milling [28]. Acetic acid and unwanted compounds are removed from the cocoa beans, and the typical baking flavor of cocoa is formed [45]. Cocoa butter, cocoa liquid, and cocoa powder are produced in this process [46]. In the next step, cocoa power, cocoa butter, milk, sugar, and other ingredients are mixed [28,37]. The residual of volatile acid is removed in the conching process to form a chocolate flavor. Temperature is controlled in the tempering process to improve the texture and quality of the chocolate. Finally, the chocolate products are packaged and distributed to retailers, and then purchased by customers [28]. During the manufacturing processes, cocoa shell and other emissions are generated. Energy consumption and pollutant emissions take place during transportation [17]. In addition, waste chocolate and packaging material are sent to incinerators or landfill, or recycled [47].

The reviewed studies conducted LCAs for different life cycle stages. Among the selected articles, 15 conducted a full LCA that considered all the life cycle stages, while other studies conducted partial LCAs to focus on certain life cycle processes. For example, Hajiyeva and Shamilova-Jalilova [48] conducted a partial LCA to study cocoa, chocolate and sugar candy in Azerbaijan from 2014 to 2020. They evaluated the production, food consumption, and usage phases of cocoa, chocolate and candy, while packaging, transportation, and waste disposal were not considered. Eighteen articles included cocoa cultivation due to its significant environmental impacts. For example, Utomo et al. [34] compared two cultivation methods of cocoa, monoculture, and agroforestry systems, and found that the cocoa–coconut cultivation system can promote environmental sustainability. The production of packaging materials also attracted attention. For instance, Büsser and Jungbluth [49] investigated aluminum foil and paper as chocolate wrappers. Sixteen of twenty-five articles argued that the manufacturing phase has the highest environmental impacts (e.g., [37]). The end-of-life stage was studied by 12 articles, whereas the waste processing of chocolate packaging was of most concern [37]. In addition, Konstantas et al. [44] not only considered packaging waste, but also addressed the recycling of waste and energy.

#### 3.2.4. Allocation

When the life cycle of a product provides other functions, the study system becomes versatile and complicated [50]. This can generate by-products and lead to waste recycling. In that case, it is necessary to conduct allocation that can address a multi-functional problem [51]. Usually, subdividing a unified process or expanding a product system cannot prevent allocation. The input and output of a product have to be allocated to reflect the physical relationship or economic values [52]. Moretti et al. [53] proposed to clarify differences between physical and other relationships for allocation. Therefore, the selection of an appropriate allocation method is crucial in LCA.

A total of 13 studies explicitly explained the allocation method, while the other 12 studies did not provide information on allocation methods. Among the 13 studies, 5 conducted allocation based on economic value [28,34,35,42,43]. It should be noted that Jeswani et al. [43] expanded the study system, as flour-making symbiotic products cannot be produced in alternative systems. In addition, Konstantas et al. [54] used a mass-based allocation method. Three articles conducted both mass- and energy-/economy-based allocation. Recanati et al. [19] first adopted mass-based allocation for cocoa shells and then adopted energy-based allocation in the manufacturing processes. Pérez Neira [55] carried out allocation based on mass and economic values between products and by-products. Parra-Paitan and Verburg [31] conducted allocation based on site feasibility, land use policy, and the capacity of lands to analyze land transformation. Raschio et al. [56] conducted allocation for inputs according to the factory site area (production per hectare per year). New allocation methods were also identified in the selected studies. Ineichen et al. [57] developed a biophysical allocation method based on the net energy demands of milk and meat production, and allocated environmental impacts according to the proportion of net energy consumed to feed lactation (milk) and growth (meat). Moreover, allocation methods should be carefully determined for an accurate and effective LCA.

### 3.3. Life Cycle Inventory

#### 3.3.1. Data Collection

The life cycle inventory (LCI), which requires a large amount of data, is the most time-consuming and data-intensive stage in LCA [58]. LCI data can be collected from four sources, namely, literature/standards, LCA tools, databases, and Environmental Product Declarations (EPDs) [59]. The reviewed articles mostly used the Sphera (GaBi) and Ecoinvent databases [60]. For example, Pérez-Neira et al. [17] integrated Ecoinvent 3.5 and Agribalyse 3.0, and conducted LCA in SimaPro 9.1.08. Ntiamoah and Afrane [61] adopted Ecoinvent and GaBi 4 to collect background data on the production, transportation, and power generation of fertilizers and pesticides. Since different databases can yield different results, it is necessary to understand the crucial differences between databases [62].

#### 3.3.2. Inputs in LCI

**Figure 6:**
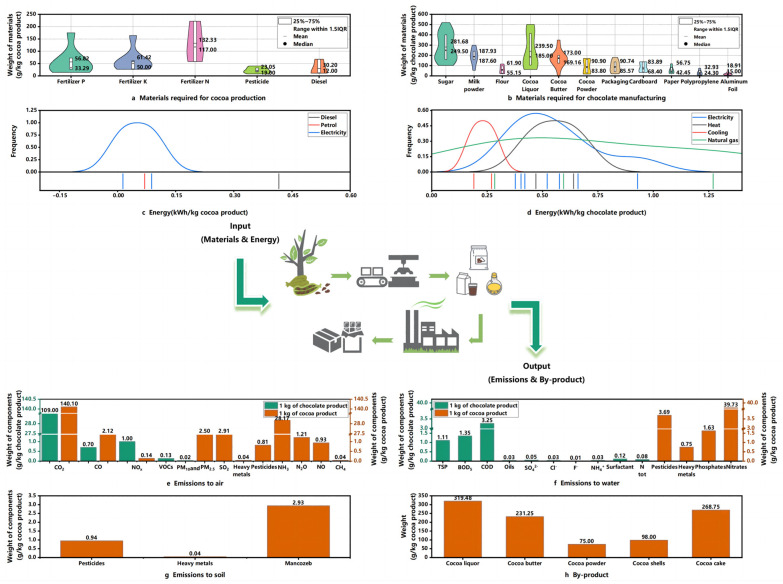
Analysis of input and output data of the chocolate industry. (**a**) Materials for cocoa production; (**b**) materials for chocolate manufacturing; (**c**) energy per kg cocoa product; (**d**) energy per kg chocolate product; (**e**) emissions to air; (**f**) emissions to water; (**g**) emissions to soil; (**h**) by-products.

The data inputs of the reviewed studies are summarized in Figure 6a–d. It is found that the materials input for cocoa production include phosphate fertilizer, potash fertilizer, nitrogen fertilizer, pesticide, and diesel. The mean (132.33 g/kg cocoa) and lower limit of nitrogen fertilizer are higher than those of other substances. On the other hand, the mean (23.05 g/kg cocoa) and lower limit of pesticides are the lowest. In the chocolate production process, the input includes sugar, milk powder, flour, cocoa liquid, and cocoa butter. It is found that the upper and lower limits of sugar, cocoa liquid, cocoa fat and milk powder vary significantly, as the contents of these ingredients vary across chocolate products. Notably, the average values of sugar, cocoa liquid and milk powder are the highest among all ingredients. As shown in Figure 6c,d, energy is mostly consumed in the chocolate production stage. Electricity is consumed in both the cocoa production and chocolate manufacturing stages. The average electricity consumption is around 0.5 kWh/kg chocolate product, indicating that chocolate manufacturing demands much electricity. The input data of the chocolate industry are summarized in Table S1 of the Supplementary Materials.

#### 3.3.3. Outputs in LCI

Output flows include wastes, emissions, and final products [63]. In Figure 6e, the emissions to air per unit of chocolate or cocoa are analyzed. It is found that both cocoa and chocolate products yield large amounts of carbon dioxide. Cocoa generated 140 gCO_2_/kg product, while chocolate generated 109 gCO_2_/kg product [19]. In addition, significant ammonia emissions were generated by cocoa (28.17 g NH_3_/kg product) [37]. Various substances can be generated and discharged into water by the chocolate industry (Figure 6f). Chemical oxygen demand (COD) can reach as high as 3.25 g/kg product [19]. In addition, the emissions of nitrates from cocoa production are 39.79 g/kg product, which is higher than other substances. The production of cocoa can also generate residual pesticides, heavy metals and mancozeb in the soil (Figure 6g), and mancozeb had the highest value at 2.93 g/kg product. By-products in the system are produced during cocoa production, including cocoa liquid (319.48 g/kg), cocoa butter (231.25 g/kg), cocoa powder (75 g/kg), cocoa shells (98 g/kg) and cocoa cakes (268.75 g/kg) [61].

#### 3.3.4. LCIA Methods

LCIA translates the LCI results into understandable indicators. Three areas of protection (AoPs) are usually considered in LCIA, namely, human health, the ecosystem, and resources [64]. In general, an LCIA method can be classified according to cause–effect chain evaluations. The midpoint method refers to an intermediate position of the cause–effect chain, while an endpoint (damage-oriented) method refers to the level of ultimate societal concern of three AoPs [65]. Many existing LCIA methods consider different impact categories and AoPs using midpoint/endpoint approaches, including CML, EDIP, Eco-indicator 99, IMPACT, IPCC, EPS, and so forth [66]. The selection of an appropriate LCIA method is essential for an LCA study [67].

**Figure 7:**
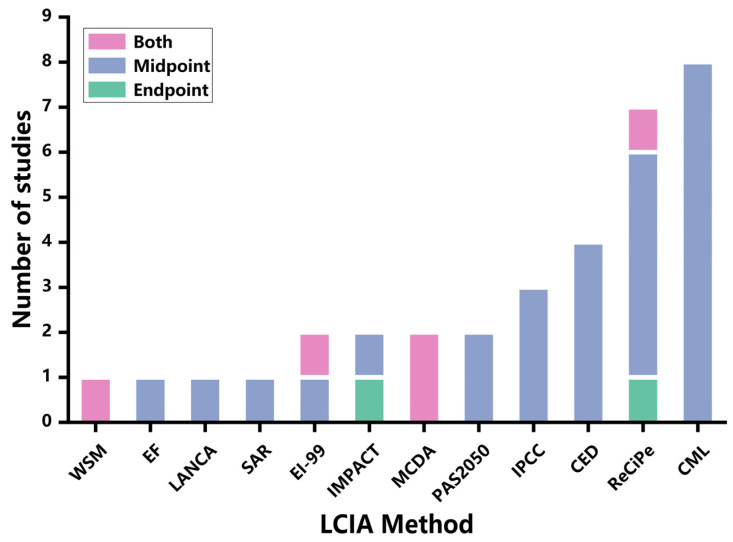
Analysis of LCIA methods of the selected studies.

Figure 7 provides an analysis of the LCIA methods adopted by the reviewed articles. It has been found that 12 LCIA methods were adopted by the LCA studies in the chocolate industry, including Eco-indicator 99, IMPACT (Impact Assessment of Chemical Toxics), MCDA (Multi-Criteria Decision Analysis), PAS2050 (British Standards Institute publicly available specification 2050), IPCC (The Intergovernmental Panel on Climate Change), CED (Cumulative Energy Demand), ReCiPe, CML (the Centre of Environmental Science-Leiden University), WSM (Weighted Sum Method), EF (Environmental Footprint), LANCA (Land Use Indicator Value Calculation in Life Cycle Assessment), and SAR (Species–Area Relationship) [47,68]. The most widely used LCIA methods are CED, ReCiPe, and CML. Nine articles applied more than one LCIA method. For example, Parra-Paitan and Verburg [31] adopted IMPACT World+ to study acidification and eutrophication, while PAS2050 was used to study the impact of land use transformation on carbon emissions. Recanati et al. [19] employed CML-IA 2001 to analyze eutrophication, ozone depletion, and acidification, while CED was used for energy demand analysis.

Most LCA studies of the chocolate industry have employed the midpoint approach. Twenty-two articles conducted midpoint analyses, while five articles conducted both midpoint and endpoint analyses. Crenna et al. [68] conducted midpoint analysis using Environmental Footprint (EF 2.0) for 16 impact categories, while ReCiPe 2016 was adopted for the endpoint analysis of biodiversity and land use. In addition, three studies did not indicate which LCIA method was employed [17,30,44].

#### 3.3.5. Impact Categories

**Figure 8:**
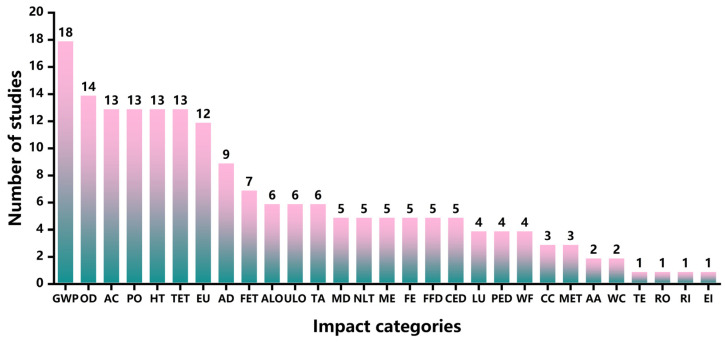
Frequency of impact categories in the selected studies. Global warming potential (GWP), ozone depletion (OD), acidification (AC), photochemical oxidation (PO), human toxicity (HT), terrestrial ecotoxicity (TET), eutrophication (EU), abiotic depletion (AD), freshwater ecotoxicity (FET), agricultural land occupation (ALO), urban land occupation (ULO), terrestrial acidification (TA), mineral depletion (MD), natural land transformation (NLT), marine eutrophication (ME), freshwater eutrophication (FE), fossil fuel depletion (FFD), Cumulative Energy Demand (CED), land use (LU), primary energy demand (PED), water footprint (WF), climate change (CC), marine ecotoxicity (MET), atmospheric acidification (AA), water consumption (WC), respiratory organics (RO), respiratory inorganics (RI), energy intensity (EI).

Figure 8 shows the impact categories studied in the selected articles. It was found that 29 impact categories were covered by the LCA studies in the chocolate industry. The most frequently studied impact category was climate change or global warming, which was covered by 18 articles. The impact category of ozone depletion was studied in 14 articles. In addition, 13 articles analyzed acidification, photochemical oxidation, human toxicity, and terrestrial ecotoxicity. On the other hand, respiratory organics, respiratory inorganics, and energy intensity (EI) were the least studied in the chocolate industry.

#### 3.3.6. LCIA Results

##### Impacts of Life Cycle Stages

Table S3 provides the environmental impacts of 1 kg chocolate product in the five major life cycle stages for the seven most-studied impact categories, including global warming, ozone depletion, acidification, photochemical oxidation, human toxicity, terrestrial ecotoxicity, and eutrophication. Büsser and Jungbluth [49] found that the upstream stages of cultivation and chocolate manufacturing accounted for 70–85% of life cycle environmental impacts, while retail packaging only accounted for 5–15% and transportation stage accounted for the minimum. In addition, Jeswani et al. [43] found that raw material accounted for 48% of the total life cycle GHG emissions, while manufacturing processes was the second largest contributor, at 23%, with packaging and transportation each accounting for 15%. Although transportation contributed relatively less than other stages, Pérez-Neira et al. [17] found that a long transport distance may lead to disadvantages for traditional chocolate as compared with organic chocolate, indicating that the impact of transportation cannot be ignored.

##### Impacts of Raw Material Production and Manufacturing

The production of raw materials and chocolate manufacturing lead to significant environmental impacts [28,69]. It has been claimed that the cultivation of cocoa in the chocolate industry causes significant deforestation, including encroachment upon protected forest areas [70]. This also leads to environmental degradation and negatively affects biodiversity, especially in open-land systems [71]. Additionally, cocoa agriculture can have adverse effects on regional runoff pollution, soil fertility, and carbon sequestration [72]. Dairy farming and milk powder production are significant contributors to carbon emissions. Escribano et al. [73] indicated that milk production alone can result in carbon emissions ranging from 1.77 to 4.09 kgCO_2_ eq/kg, with the gastrointestinal fermentation and feeding processes of livestock being the primary influencing factors. In addition, during the spray-drying process of milk powder production, steam in the primary energy demand (PED) accounted for 49.99–90.55% of energy and resource consumption, while the electricity accounted for 9.43–49.99% [74]. The production of chocolate consumes considerable water, electricity, and heat, which have adverse effects on the climate [54]. The manufacturing processes also pose risks to human and ecological health due to chemical emissions, and impact the environment through ozone layer depletion, eutrophication, and acidification [28].

**Figure 9:**
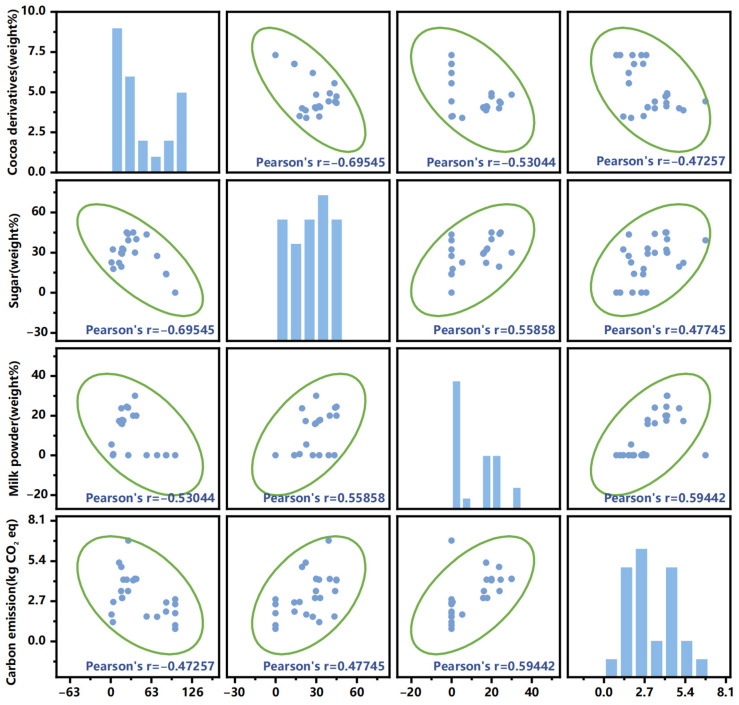
Distribution and correlation analysis of life cycle carbon emissions and dosages of three ingredients (cocoa derivatives, sugar, and milk powder) of chocolate from the selected articles. Twenty-five datasets from 13 articles are analyzed [17,19,28,35,36,37,43,44,47,48,49,54,55].

Figure 9 shows the correlation between three ingredients and the life cycle carbon emissions of different chocolate products studied in the selected articles. It is found that carbon emissions are positively correlated with dosages of milk powder and sugar, with Pearson’s r values of 0.594 and 0.477, respectively. On the other hand, the dosage of cocoa derivatives is negatively correlated with carbon emissions, with a Pearson’s r value of −0.472. Konstantas et al. [54] also found that increasing the dosage of milk can lead to increased environmental impacts of chocolate products. In addition, Miah et al. [36] explored the carbon emissions of chocolate confectionery, and found that dark chocolate confectionery with 39.1% sugar led to greater carbon emissions than milk chocolate confectionery (22.2% sugar) and milk chocolate biscuit confectionery (19.5% sugar).

The distributions of the three ingredients and carbon emissions are provided in Figure 9. It is found that most of the selected articles demonstrated a milk powder proportion of between 15% and 25%. The proportions of cocoa derivatives were mostly distributed between 10% and 30%. In certain cases, for example, dark chocolate with 14% sugar and 86% cocoa derivatives, but no milk powder, can emit 2 kg CO_2_ eq/kg product [37]. Pérez-Neira et al. [17] studied pure chocolate with 100% cocoa derivatives and found that the carbon emission was as low as 1.9 kg CO_2_ eq/kg product.

##### Comparison of Different Types of Chocolate Product

The comparison of carbon emissions of different types of chocolate products is performed according to three categories, namely, chocolate recipe (e.g., dark chocolate, white chocolate), manufacturing and packaging methods, and cultivation management (Table S4). It is found that the life cycle carbon emissions involved in producing 1 kg of chocolate product range from 1.29 to 6.76 kg CO_2_ eq. Manufacturing and packaging methods consider molded chocolate, chocolate countlines, and chocolate in a bag. Chocolate countlines made the largest contribution to the total impact (37–43%), followed by chocolates in a bag (28–33%) [54]. The primary emission source of packaging is related to the acquisition of material for packaging and waste management [75]. It is necessary to replace plastic packaging with cassava starch and sugarcane [76], or with bioplastics [77]. Concerning the cultivation of cocoa, the carbon emission of 1 kg traditional cultivated cocoa beans is 6 kg CO_2_ eq lower than that of technically cultivated cocoa beans [33]. In addition, cocoa agroforestry performs better than cocoa monoculture [31,78]. While the traditional method performs better than the technical method in terms of carbon emissions, other factors, such as economic efficiency, time consumption, working conditions, etc., should be incorporated when selecting the cultivation method.

**Figure 10:**
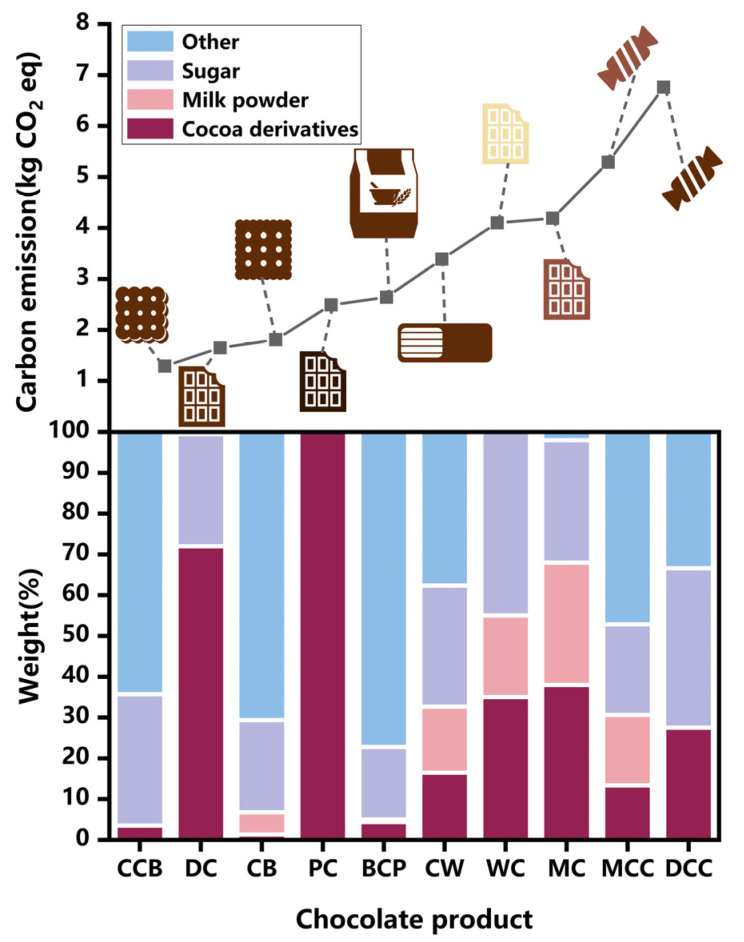
Ranking of chocolate product types in terms of carbon emissions. (CCB—chocolate cream biscuit, DC—dark chocolate, CB—chocolate-coated biscuit, PC—pure chocolate, BCP—breakfast cereal products, CW—chocolate wafer, WC—white chocolate, MC—milk chocolate, MCC—milk chocolate confectionery, DCC—dark chocolate confectionery).

Figure 10 gives a ranking of carbon emissions for chocolate product types. It is found that chocolate cream biscuits had the lowest emissions of 1.29 kg CO_2_ eq/kg product [79], while chocolate confectionery had the highest emissions of 6.76 kg CO_2_ eq/kg product [36]. The emissions from dark chocolate are relatively lower (1.67 kg CO_2_ eq/kg product) [28], compared to white and milk chocolate (4.1 and 4.19 kg CO_2_ eq/kg product) [28,37]. This is due to the large proportion of milk in white and milk chocolate. The emission factor of milk powder is 7.4 kg CO_2_ eq/kg milk powder [80], due to the large emissions generated via dairy farming [28,37,49]. On the other hand, the emission factor of cocoa derivatives ranges from 1.0 to 4.3 kg CO_2_ eq/kg [61], which is much smaller than that of milk powder. Therefore, dark chocolate that contains more cocoa derivatives but no milk has lower emissions. In addition, the emission factors of sugar are very small, ranging from 0.45 to 0.63 kg CO_2_ eq/kg [81].

##### Reduction in GHG Emissions of the Chocolate Industry

In response to the demand for GHG reduction, the selected articles conducted in-depth analyses of the chocolate industry. For the raw material stage, GHG emissions caused by milk production can be reduced by improving feed for cows, and reusing manure as a fertilizer for crops [82]. The combustion of sugarcane residues can cause carbon emissions [83], which can be reduced by establishing a green harvest system and using bagasse as a replacement for fossil fuel in sugar mills [84]. In addition, cocoa–coconut agroforestry [34] can significantly reduce the GHG emissions produced during cocoa cultivation.

### 3.4. Interpretation

#### 3.4.1. Sensitivity Analysis

Sensitivity analysis is a crucial step in ensuring the robustness of results. Among the selected studies, eight conducted sensitivity analyses. Konstantas et al. [54] studied the influences of land transformation, raw material loss, and data sources. Bianchi et al. [37] conducted sensitivity analysis on the allocation of energy consumption of cocoa byproducts. Additionally, Büsser and Jungbluth [49] carried out sensitivity analyses on alternative scenarios of shopping and cooling methods, and found that the shopping scenario, with its greater traffic demand, increases the environmental impact. These studies conducted sensitivity analyses on the consumption of materials and energy, as well as usage scenarios, based on the characteristics of their products and research purpose, making their conclusions more reliable. However, it is evident that the number of studies performing sensitivity analyses is insufficient, highlighting the importance of including this aspect in future research to provide a comprehensive illustration.

#### 3.4.2. Uncertainty Analysis

As an environmental impact assessment tool, LCA may be hindered by numerous uncertainties related to calculation, technology, and data source, which pertain to the quantification and dissemination of the entire process from input uncertainty to output uncertainty. Therefore, to enhance the reliability of the LCA results, establish credibility, and prevent errors, conducting the uncertainty analysis of models and results is essential. Among the selected studies, four conducted uncertainty analyses. For example, Konstantas et al. [44] performed Monte Carlo simulations with @RISK 7 [85] to investigate the PERT distribution [86] for each variable. They found that the results for chocolates in bags and chocolate premium ice cream can vary by ±12%, while results for low-fat or -sugar biscuits can vary by ±19%. Jeswani et al. [47] also performed Monte Carlo simulation using the RiskAMP add-in to study the uncertainty caused by land use inventory and biodiversity. However, the lack of uncertainty analyses performed in the other studies can impact the accuracy of the process and results.

#### 3.4.3. Research Findings of Literature

The research findings from the selected studies are summarized in Table S5 in accordance with the six research aims discussed in Section 3.2.1. The raw material production stage and chocolate manufacturing stage have the greatest environmental impacts, accounting for 70–85% of total carbon emissions [49]. Milk powder and cocoa derivatives contribute significantly in these two stages. The cocoa derivatives in dark chocolate contributed up to 91% and 96% to eutrophication potential (EP) and acidification potential (AP), and milk powder in white chocolate contributed 76.3% and 65.1% to EP and AP, respectively [37]. In addition, energy consumption (diesel, gasoline and oil) is the key contributor in both production and manufacturing phases, and is responsible for about 66.5% and 16.1% of the entire life cycle’s GHG emission [55]. Packaging and transport processes, being the smallest contributors, account for 5–20% of the GHG emissions for the entire life cycle [49].

#### 3.4.4. Integration with Other Techniques

In the reviewed studies, the integration of LCA with other research techniques was conducted to improve the research accuracy. Parra-Paitan and Verburg [31] integrated land use modeling to conduct spatial analyses. Raschio et al. [56] performed coupled geospatial analysis through geographic information systems (GIS) to calculate GHG emissions. Parra-Paitan and Verburg [31] adopted the CLMondo model [87] to simulate land use changes, and integrated it with the GLOBIO-InVEST model [88] to calculate biodiversity changes.

## 4. Discussion

### 4.1. Opportunities of LCA in the Chocolate Industry

In the whole life cycle of chocolate, the production stage of raw materials and the product manufacturing stage have the greatest impacts, and the planting management mode of cocoa beans and other raw materials will significantly affect the yield, as well as the corresponding environmental impacts [29]. Therefore, it is necessary to clarify the variety and farming stage of raw materials when evaluating the environmental impact of the planting stage. Clearly evaluating the energy and material input during the manufacturing, as well as the emissions, remains challenging for LCA [89]. The variety of chocolate products is complex, and the environmental effects of different recipes vary considerably. The proportion of cocoa derivatives, milk powder, and sugar in products has a great relationship with carbon emissions. Therefore, it is necessary to apply LCA to analyze the influential factors of environmental impacts and provide suggestions for the design of formulations. In addition, LCA can provide low-carbon and environmentally friendly strategies for the clean production and selection of packaging materials for chocolate and other products [90]. Since raw materials are extracted from countries all over the world, and the factories and sales locations are different, the mode of transportation will change for different life cycles. Therefore, transportation details need to be clear to ensure accurate results. The waste treatment stage mainly involves different treatments of product packaging, which also produces different impact results [91].

LCA has been implemented in the chocolate industry for fifteen years. However, there are still many limitations in this field. Currently, LCA studies of the industry can be mostly found in Europe, with few studies in Asia, South America and Africa. In addition, research is still lacking in regions of Oceania and North America. Chocolate is a popular product across the world, and more case studies are needed in those regions with a lack of studies. Uncertainty analysis and sensitivity analysis are essential steps in LCA. However, not many studies conducted these two analyses. Future research on the LCA of the chocolate industry should involve uncertainty analysis and sensitivity analysis. For the chocolate industry, it is recommended to further improve the manufacturing and cultivation processes, which have the largest contributions. It is also necessary to conduct LCAs for each chocolate design to select an environmentally friendly option with equal flavor, function, and quality. In addition, impacts related to packaging material, waste treatment and transportation cannot be ignored and need further attention.

Based on the conclusions of chocolate LCA research, it can be inferred that most studies have emphasized the impacts of chocolate on global warming. Although there have been discussions on other environmental impact categories, they are not sufficient. In future research, it is necessary to not only deepen the exploration of different impact categories, but also to normalize the results of environmental impacts to obtain more comprehensive conclusions, which is more conducive to the progress of the industry. In addition, the reviewed LCA studies conducted environmental assessments for certain countries, regions, or products, which can obtain more accurate inventory data and analysis results, providing corresponding improvement suggestions for specific problems. However, there is a lack of exploration into the overall global environmental impact of chocolate. It is recommended that future research should broaden this horizon by conducting more comprehensive and insightful discussions of the environmental issues associated with the chocolate industry.

### 4.2. Limitations of This Study

In this study, a literature review on 25 LCA studies in the chocolate industry is conducted. The lack of case studies is one of the key limitations of this review. This analysis being based on limited samples may lead to bias in the results. In addition, as the system boundaries, data sources, and methods are different in the selected studies, the differences in results within the studies may not be caused by the actual differences in chocolate products, but may be generated by the different methods, data, or analysis scopes.

## 5. Conclusions

Based on the systematic review approach, this study reviewed and analyzed 25 existing articles on chocolate LCA. It investigated the most advanced LCA applications in the current chocolate industry, and analyzed and summarized the data inventory and evaluation results in the literature. After comparing the environmental performances of different categories of chocolate products using qualitative and quantitative methods, suggestions were made to improve the future sustainability of the chocolate industry.

It is found that the environmental impact of the chocolate industry has received attention in recent years, but there is lack of application of LCAs. Throughout the life cycle of chocolate, raw material production and chocolate manufacturing have the greatest environmental impact, accounting for 70–85%. The former produces the highest carbon emissions, reaching 1.1 kg of carbon dioxide equivalent per kilogram. Transportation, waste treatment, and other stages have been ignored by many studies because of missing data or relatively lower environmental impacts, and only 15 articles involved all life cycle stages. Cocoa derivatives, milk powder, and sugar are the main factors causing carbon emissions, and the correlations vary in different products. Among the chocolate products in the studies, dark chocolate has a much lower environmental impact (1.67 kg CO_2_ eq/kg product) than white chocolate (4.1 kg CO_2_ eq/kg product) and milk chocolate (4.19 kg CO_2_ eq/kg product). For products containing chocolate ingredients, biscuits have the lowest GHG emissions (1.29 kg CO_2_ eq/kg product for chocolate cream biscuit, 1.81 kg CO_2_ eq/kg product for chocolate-coated biscuit), while chocolate confectionery products produce the highest carbon emissions (5.29 kg CO_2_ eq/kg milk chocolate confectionery and 6.76 kg CO_2_ eq/kg dark chocolate confectionery).

Therefore, the application of LCA methods in the industry needs to be further promoted in the future. LCA research on chocolate should establish a complete system boundary based on the research purpose and select the appropriate functional units according to the nature and function of the product. Combining traditional and new methods during the assessment also benefits the research analysis. In addition, performing uncertainty analysis and sensitivity analysis at the end of the study is important if we are to improve the accuracy and reliability of LCA results for chocolate products. Sustainable management during the material production stage, as well as the formulation of the design of the products, is also noteworthy for its capacity to reduce the environmental impacts of chocolate. In the future, innovatively combining LCA with new methods and investigating the best formula based on the balance between the product quality and environmental impact will be an opportunity and a challenge for the chocolate industry.

## Data Availability

The original contributions presented in the study are included in the article/supplementary material, further inquiries can be directed to the corresponding author.

## Supplementary Materials

The following supporting information can be downloaded at: https://www.mdpi.com/article/10.3390/foods13060915/s1, Table S1: The input data within the boundaries of the chocolate system, Table S2: The output data within the boundaries of the chocolate system, Table S3: Environmental impacts of 1 kg chocolate product, Table S4: Comparison of different types of chocolate product, Table S5: Summary of research findings of reviewed articles.

## Appendix A

**Table A1:** Summary of selected studies on LCA in the chocolate industry.

Author (Year)	Object	Functional Unit	Life Cycle Stages
Ntiamoah and Afrane (2008) [61]	Cocoa	1 kg of cocoa beans processed	Cradle to grave
Büsser and Jungbluth (2009) [49]	Dark, milk and white chocolate and chocolate with sultanas	1 kg of chocolate	Cradle to retailer
Ortiz-R et al. (2014) [27]	Cocoa	1 ha of land planted with cocoa (25 year life span)	Cradle to grave
Jeswani et al. (2015) [43]	Breakfast cereals and snacks	1 kg of breakfast cereal products	Cradle to grave
Ortiz-Rodríguez et al. (2016) [33]	Cocoa	1 kg of cocoa beans	Cradle to farm gate
Pérez Neira (2016) [55]	Chocolate	1 kg of pure chocolate	Cradle to retailer
Utomo et al. (2016) [34]	Cocoa pod	1 t of cocoa pod	Cradle to on-farm gate
James et al. (2017) [30]	Cocoa	1 ton of dried cocoa beans	Gate to gate
Miah et al. (2018) [36]	Sugar, milk chocolate, dark chocolate, chocolate biscuit and milk	1 kg of packaged confectionery product	Cradle to grave
Konstantas et al. (2018) [54]	Chocolate coated wafers (chocolate countlines), milk chocolate (molded chocolate) and malty chocolates (chocolates in bag)	1 kg of packaged chocolate consumed at home	Cradle to grave
Recanati et al. (2018) [19]	Italian dark chocolate	1 kg of dark chocolate and packaging	Cradle to grave
Jeswani et al. (2018) [47]	Breakfast cereals and snacks	The annual production of ready-to-eat breakfast cereal products	Cradle to grave
Raschio et al. (2018) [56]	Cocoa	1 hectare per year of land to produce cocoa; 1 t of raw cocoa	Farming stage
Konstantas et al. (2019) [44]	Biscuits, cakes, chocolates and ice creams	1 kg of product consumed at home; The annual consumption of each product in the UK	Cradle to grave
Konstantas et al. (2019) [42]	Ice cream with differing compositions and flavors (vanilla regular; vanilla premium; chocolate regular; and chocolate premium)	1 kg of ice cream consumed at home	Cradle to grave
Konstantas et al. (2019) [35]	Six types of biscuit: crackers, low fat/sugar, semi-sweet, chocolate-coated, chocolate cream sandwich and vanilla cream sandwich	1 kg of biscuits consumed at home	Cradle to grave
Crenna et al. (2019) [68]	32 representative food products of consumption in the European Union	The average food consumption in the European Union and per EU citizen in terms of selected food categories in 2015	Cradle to grave
Pérez-Neira et al. (2020) [17]	Ecuadorian cacao/chocolate	1 kg of dark chocolate	Cradle to retailer
Bianchi et al. (2021) [37]	Dark, milk and white chocolate	1 kg of chocolate; 1 kcal	Cradle to grave
Konstantas et al. (2020) [79]	Biscuits, cakes, chocolates and ice creams	1 kg of packaged product consumed at home; the annual consumption based on the sales of the products in the two sectors	Cradle to grave
Armengot et al. (2021) [29]	Cocoa	1 kg of cacao; 1 kg of cocoa, banana and other crops	Cradle to farm gate
Boakye-Yiadom et al. (2021) [28]	Extra dark chocolate (EDC), dark chocolate (DC), milk chocolate (MC), and flavoured milk chocolate (FMC)	1 kg packaged chocolate bar	Cradle to retailer
Hajiyeva and Shamilova-Jalilova (2022) [48]	Cocoa, chocolate and sugar confectioneries	1 kg of the product	Gate to gate
Caicedo-Vargas et al. (2022) [38]	Cocoa	1 kg of cocoa; 1 kg of output sold; 1 ha	Cradle to farm gate
Parra-Paitan and Verburg (2022) [31]	Cocoa	1 kg of cocoa beans	Around the agricultural phase
