# Supplementary material for: Applications of Life Cycle Assessment in the Chocolate Industry: A State-of-the-Art Analysis Based on Systematic Review
Source: Foods. 2024 Mar 18;13(6):915. doi: 10.3390/foods13060915 (PMC10969383; doi:10.3390/foods13060915)
Supplement: Supplementary file 1 [file foods-13-00915-s001.zip › foods-2901480-supplementary.pdf]

Applications of Life Cycle Assessment in the Chocolate Industry: A State-of-the-Art Analysis Based on Systematic Review

Supplementary Material

Shuhan Wang[1], Yahong Dong[1,2*]

1 Macau Environmental Research Institute, Macau University of Science and Technology, Macao, China

2 National Observation and Research Station of Coastal Ecological Environments in Macao, Macau University of Science and Technology, Macao, China

* Correspondence: yhdong@must.edu.mo

Table S1. The input data within the boundaries of the chocolate system.

Input data			
**Energy**	**Amount (per kg chocolate product)**	**Unit**	**Data sources**
Electricity (trigeneration)	0.42—0.91	kWh	(Recanatia et al., 2018; Bianchi et al.,2021; Jeswani et al, 2015) [19,37,43]
Heat (trigeneration)	0.47—0.64	kWh	(Recanatia et al., 2018; Bianchi et al.,2021) [19,37]
Cooling (trigeneration)	0.19—0.27	kWh	(Recanatia et al., 2018; Bianchi et al.,2021) [19,37]
Electricity (national grid)	0.019	kWh	(Recanatia et al., 2018) [19]
Natural gas (national grid)	1.023—4.57	MJ	(Recanatia et al., 2018; Bianchi et al.,2021; Jeswani et al, 2015) [19, 37,43]
Electricity	0.37—0.57	kWh	(Konstantas et al., 2019a; Konstantas et al., 2019c) [44,35]
**Energy**	**Amount (per kg cocoa product)**	**Unit**	**Data sources**
Electricity (national grid)	0.088	kWh	(Ntiamoah and Afrane, 2008) [61]
Electricity	0.014	kWh	(Leyte et al., 2017) [30]
Diesel	53.14	g	(Ntiamoah and Afrane, 2008) [61]
Petrol	8.99	g	(Ntiamoah and Afrane, 2008) [61]
**Materials**	**Amount (per kg chocolate product)**	**Unit**	**Data sources**
Sugar	102.2—520	g	(Recanatia et al., 2018; Boakye-Y et al., 2021; Bianchi et al.,2021; Miahab et al., 2018; Konstantas et al., 2019a; Konstantas et al., 2019b; Jeswani et al., 2015; Konstantas et al., 2019c) [19,28,37,36,44,42,43,35]
Milk powder	0—300	g	(Boakye-Y et al., 2021; Bianchi et al.,2021; Konstantas et al., 2019a; Konstantas et al., 2019c) [28,37,44,35]
Flour	21.71—115.6	g	(Konstantas et al., 2018; Konstantas et al., 2019a; Jeswani et al., 2015; Konstantas et al., 2019c) [54,44,43,35]
Butter	2.38—2.4	g	(Konstantas et al., 2018;Konstantas et al., 2019a) [54,44]
Starch	21.71—23.4	g	(Konstantas et al., 2018;Konstantas et al., 2019a) [54,44]
Packaging	20—180.77	g	(Recanatia et al., 2018; Konstantas et al., 2018; Neira, 2016; Bianchi et al., 2021) [19,54,55,37]
Aluminum Foil	6.6—39.8	g	(Recanatia et al., 2018; Konstantas et al., 2018; Pérez-N et al., 2020; Neira, 2016; Boakye-Y et al., 2021; Miahab et al., 2018; Konstantas et al., 2019a; Crenna et al., 2019) [19,54,17,55,28,36,44,68]
Cardboard	33—137	g	(Recanatia et al., 2018; Konstantas et al., 2018; Bianchi et al., 2021; Konstantas et al., 2019a; Crenna et al., 2019; Konstantas et al., 2019b; Konstantas et al., 2019c) [19,54,37,44,68,42,35]
Paper	24.1—118	g	(Pérez-N et al., 2020; Neira, 2016; Bianchi et al., 2021; Boakye-Y et al., 2021) [17,55,37,28]

Materials	Amount (per kg cocoa product)	Unit	Data sources
Polypropylene	8.1—75	g	(Bianchi et al., 2021; Miahab et al., 2018; Konstantas et al., 2019b; Konstantas et al., 2019c) [37,36,42,35]
Fertilizer N	58—222	g	(Bianchi et al., 2021) [37]
Fertilizer P	14—175	g	(Bianchi et al., 2021; Parra-P and Verburg, 2022; Crenna et al., 2019) [37,31,68]
Fertilizer K	26.27—164	g	(Bianchi et al., 2021; Parra-P and Verburg, 2022; Crenna et al., 2019) [37,31,68]
Organic fertilizer	15—21	g	(Bianchi et al., 2021) [37]
Pesticide	8.23—40	g	(Bianchi et al., 2021; Crenna et al., 2019) [37,68]
Diesel	11—67.6	g	(Bianchi et al., 2021; Leyte et al., 2017) [37,30]
Petrol	0.019—0.024	L	(Parra-P and Verburg, 2022) [31]

Table S2. The output data within the boundaries of the chocolate system.

Output data			
**Emissions to air**			
**Component**	**Amount (per kg chocolate product)**	**Unit**	**Data sources**
CO_2	109	g	(Recanatia et al., 2018) [19]
VOCs	128.9	mg	(Recanatia et al., 2018) [19]
PM_{10} and $PM_{2.5}$	22.6	mg	(Recanatia et al., 2018) [19]
NOx	1	g	(Recanatia et al., 2018) [19]
CO	0.7	g	(Recanatia et al., 2018) [19]
**Component**	**Amount (per kg cocoa product)**	**Unit**	**Data sources**
CO_2	44.11—237.9	g	(Ntiamoah and Afrane, 2008; Leyte et al., 2017; Parra-P and Verburg, 2022) [61,30,31]
PM_{10} and $PM_{2.5}$	2.5	g	(Ntiamoah and Afrane, 2008) [61]
NOx	0.08-0.18	g	(Leyte et al., 2017; Parra-Pand Verburg, 2022) [30,31]
CO	0.018—8.41	g	(Ntiamoah and Afrane, 2008; Leyte et al., 2017; Parra-P and Verburg, 2022) [61,30,31]
SO_2	0.001—8	g	(Ntiamoah and Afrane, 2008; Leyte et al., 2017; Parra-P and Verburg, 2022) [61,30,31]
Heavy metals	0.035	g	(Ntiamoah and Afrane, 2008) [61]
Pesticides	0.813	g	(Ntiamoah and Afrane, 2008) [61]
NH_3	2.2—54	g	(Bianchi et al.,2021) [37]
N_2O	0.01—3.5	g	(Bianchi et al.,2021; Leyte et al., 2017; Parra-P and Verburg, 2022) [37,30,31]
NO	0.4—1.6	g	(Bianchi et al.,2021) [37]
CH_4	0.01—0.053	g	(Leyte et al., 2017; Parra-P and Verburg, 2022) [30,31]
**Emissions to water**			
**Component**	**Amount (per kg chocolate product)**	**Unit**	**Data sources**
TSP	1.11	g	(Recanatia et al., 2018) [19]
BOD_5	1.35	g	(Recanatia et al., 2018) [19]
COD	3.25	g	(Recanatia et al., 2018) [19]
Oils	31.68	mg	(Recanatia et al., 2018) [19]
SO_4^{2-}	53.97	mg	(Recanatia et al., 2018) [19]
Cl^-	32.85	mg	(Recanatia et al., 2018) [19]
F^-	9.5	mg	(Recanatia et al., 2018) [19]
NH_4	30.27	mg	(Recanatia et al., 2018) [19]
Surfactant	115.43	mg	(Recanatia et al., 2018) [19]
N tot	84.48	mg	(Recanatia et al., 2018) [19]
**Component**	**Amount (per kg cocoa product)**	**Unit**	**Data sources**
Nitrates	0.0175—0.0667	kg	(Ntiamoah and Afrane, 2008; Bianchi et al.,2021) [61,37]
Phosphates	0.0004—0.0027	kg	(Ntiamoah and Afrane, 2008; Bianchi et al.,2021) [61,37]
Pesticide	3.6880E−03	kg	(Ntiamoah and Afrane, 2008) [61]
Heavy metals	7.4761E−04	kg	(Ntiamoah and Afrane, 2008) [61]
**Emissions to soil**			
**Component**	**Amount (per kg cocoa product)**	**Unit**	**Data sources**
Pesticides	9.4477E−04	kg	(Ntiamoah and Afrane, 2008) [61]
Heavy metals	4.1870E−05	kg	(Ntiamoah and Afrane, 2008) [61]
Mancozeb	0.0003—0.00556	kg	(Leyte et al., 2017; Crenna et al., 2019) [30,68]
**By-products**			
**Component**	**Amount (per kg cocoa product)**	**Unit**	**Data sources**
Cocoa liquor	319.48	g	(Ntiamoah and Afrane, 2008) [61]
Cocoa butter	231.25	g	(Ntiamoah and Afrane, 2008) [61]
Cocoa powder	75	g	(Ntiamoah and Afrane, 2008) [61]
Cocoa shells	98	g	(Ntiamoah and Afrane, 2008) [61]
Cocoa cake	268.75	g	(Ntiamoah and Afrane, 2008) [61]

Table S3. Environmental impacts of 1 kg chocolate product.

Impact category	Production	Manufacturing	Packaging	Transportation	Distribution	Total	Unit	Data source
Global warming potential	1.1	0.158—0.549	0.341-0.270	0.219—1.84	0.1	1.20—4.21	kg CO_2 eq	(Recanatia et al., 2018; Pérez-N et al., 2020; Konstantas et al., 2020; Boakye-Yet al., 2021; Konstantas et al., 2019a; Konstantas et al., 2019b; Crenna et al., 2019) [19,17,79,28,44,42,68]
Ozone depletion	3.60E-07	2.69E-08—6.89E-08	1.88E-08—2.37E-08	4.14E-08—5.32E-07	1.26E-08	8.3E-08—5.5E-06	kg CFC-11 eq	(Recanatia et al., 2018; Pérez-N et al., 2020; Konstantas et al., 2020; Boakye-Y et al., 2021; Konstantas et al., 2019a; Jeswani et al., 2015; Konstantas et al., 2019b; Crenna et al., 2019) [19,17,79,28,44,43,42,68]
Acidification	0.014	2.59E-04—4.21E-03	1.70E-03—2.15E-03	1.15E-03—7.69E-03	7.66E-04	0.0054—0.0264	kg SO_2 eq	(Recanatia et al., 2018; Pérez-N et al., 2020; Boakye-Y et al., 2021; Crenna et al., 2019) [19,17,28,68]
Photochemical oxidation	2.70E-04	6.16E-05—1.59E-04	1.08E-04—1.36E-04	8.27E-05—5.54E-04	2.89E-05	3.07E-04—9.4E-03	kg C_2H_4 eq	(Recanatia et al., 2018; Pérez-N et al., 2020; Konstantas et al., 2020; Boakye-Y et al., 2021; Konstantas et al., 2019a; Jeswani et al., 2015; Konstantas et al., 2019b) [19,17,79,28,44,43,42]
Human toxicity	-	0.198	0.27	0.166—1.12	0.0361	0.41—1.81	kg 1,4-DB eq	(Pérez-N et al., 2020; Konstantas et al., 2020; Boakye-Y et al., 2021; Konstantas et al., 2019a; Jeswani et al., 2015; Konstantas et al., 2019b) [17,79,28,44,43,42]
Terrestrial ecotoxicity	-	1.78E-03	1.93E-03	9.36E-04—6.32E-03	3.25E-04	2.60E-04—0.0467	kg 1,4-DB eq	(Pérez-N et al., 2020; Konstantas et al., 2020; Boakye-Y et al., 2021; Konstantas et al., 2019a; Konstantas et al., 2019b) [17,79,28,44,42]
Eutrophication	0.0235	1.69E-04—8.28E-04	4.68E-04—5.91E-04	3.24E-04—2.21E-03	1.51E-04	0.0017—0.0255	kg PO_4^{3-}eq	(Recanatia et al., 2018; Pérez-N et al., 2020; Boakye-Y et al., 2021; Jeswani et al., 2015) [19,17,28,43]

Note: '-': not available.

Table S4. Comparison of different types of chocolate product

Product type	Impact category	Results	Unit	System boundary	Authors (year)
**Category 1: Chocolate recipe**					
Dark chocolate	GWP	2.1-2.62	kg CO_2 eq	Whole life cycle; Whole life cycle except retail stage; Whole life cycle except waste management stage; Whole life cycle except waste management stage;	(Recanatia et al., 2018; Bianchi et al., 2021; Boakye-Y et al., 2021; Büsser et al., 2009) [19,37,28,49]
Milk chocolate	GWP	3.6-4.2	kg CO_2 eq	Whole life cycle except retail stage; Whole life cycle except waste management stage; Whole life cycle except waste management stage	(Bianchi et al., 2021; Boakye-Y et al., 2021; Büsser et al., 2009) [37,28,49]
White chocolate	GWP	4.1	kg CO_2 eq	Whole life cycle except retail stage; Whole life cycle except waste management stage	(Bianchi et al., 2021; Büsser et al., 2009) [37,49]
Pure chocolate	GWP	2.57-4.15	kg CO_2 eq	Whole life cycle; Whole life cycle except waste management stage	(Konstantas et al., 2018; Neira, 2016) [54,55]
Chocolate confectionery	GWP	5.29-6.76	kg CO_2 eq	Whole life cycle;	(Miah et al., 2018) [36]
Chocolate biscuit	GWP	1.29-5	kg CO_2 eq	Whole life cycle; Whole life cycle	(Miah et al., 2018; Konstantas et al., 2019a) [36,44]
Chocolate wafer	GWP	3.39	kg CO_2 eq	Whole life cycle	(Konstantas et al., 2018) [54]
Chocolate icream	GWP	3.66-3.91	kg CO_2 eq	Whole life cycle	(Konstantas et al., 2019b) [42]
Chocolate cereal and snack	GWP	2.64	kg CO_2 eq	Whole life cycle	(Jeswani et al., 2015) [43]
**Category 2: Manufacturing and packaging methods**					
**Product type**	**Impact category**	**Results**	**Unit**	**System boundary**	**Authors (year)**
Moulded chocolate	GWP	4.15	kg CO_2 eq	Whole life cycle	(Konstantas et al., 2018) [54]
Chocolate countlines	GWP	3.39	kg CO_2 eq	Whole life cycle	(Konstantas et al., 2018) [54]
Chocolates in bags	GWP	2.91	kg CO_2 eq	Whole life cycle	(Konstantas et al., 2018) [54]
**Category 3: Cultivation management approach**					
**Product type**	**Impact category**	**Results**	**Unit**	**System boundary**	**Authors (year)**
Traditional chocolate	GWP	2.57	kg CO_2 eq	Whole life cycle except waste management stages	(Neira, 2016) [55]
Technified chocolate	GWP	2.82	kg CO_2 eq	Whole life cycle except waste management stages	(Neira, 2016) [55]

Table S5. Summary of research findings of reviewed articles.

Aim	Findings
**A1: Whole life cycle of chocolate**	▪ A full life cycle evaluation is essential for the environmental impact assessment and sustainability development of chocolate products.<br>▪ The raw material planting stage and the chocolate manufacturing stage contribute the most to the environmental impact.<br>▪ The environmental impact of the transportation stage is minimal, but through each life cycle stage, the total environmental impact on chocolate cannot be ignored.
**A2: Main environmental hotspots**	▪ Cocoa derivatives as well as milk powder are the largest contributors to the production stage.<br>▪ Energy consumption is the biggest environmental influencing factor in the manufacturing stage.<br>▪ GWP is the hot spot influence analogy, in which the raw material composition has the largest proportion.<br>▪ CED was the category where chocolate affecting the largest results.
**A3: Product comparison**	▪ According to the difference in the formula, milk chocolate has the greatest environmental impact, and dark chocolate has the lowest impact. Of all the chocolate products, chocolate confectionery makes the most significant contribution to the environmental impact.<br>▪ Chocolate products produced under traditional farm management methods and the cocoa agroforestry system are more sustainable.
**A4: Comparison of designs**	▪ Different designs of cocoa production management can produce different environmental impact results.<br>▪ The design of cocoa production management includes: cacao agroforestry systems; conventionally managed systems; technified management systems; monocultures.<br>▪ The weight of the impact of transportation is also different in different supply chain scenarios.
**A5: Incomplete life-cycle environmental impact**	▪ Some of the literature focuses on the environmental impact of the planting stage of cocoa.<br>▪ Due to the small proportion of the transportation stage, some literature ignores the impact caused by transportation.<br>▪ Some literature ignores waste management or waste and by-products statistics due to missing data or too small numbers.
**A6: New ways to combine with the life cycle assessment**	▪ LCA method combining economic evaluation method and geographic information technology can obtain the environmental and economic benefits of chocolate products.<br>▪ The use of Taguchi Loss Function measures the economic impact of a process deviation from production targets.<br>▪ Shape files that calculate GWP100 can be analyzed using geospatial analysis and GIS.<br>▪ Combine land use modeling such as CLUMondo model and spatial analysis such as GLOBIO-InVEST model to explain effects outside the farm level.
